# Clinical Lecture on Whooping Cough

**Published:** 1854-11

**Authors:** Robert B. Todd


### CLINICAL LECTURE ON HOOPING-COUGH.

BY ROBERT B. TODD, M. D., F. R. S.

There is one form of cough so peculiar and so characteristic, that it must be dealt with *per se*, constituting as it does, not alone a symptom, but in truth a disease. The hooping-cough, so called from its peculiar final whoop, which is a sign that the patient is again taking breath, is not often brought under our notice in the wards of a hospital. It is much more common among the out-patients of a hospital than among the in-patients.

Of the two examples of hooping-cough now in the hospital, one case is that of a girl, named Elizabeth Griffin, who was admitted into Lonsdale Ward, on Dec. 21, 1852 ; the other is a brother of this patient, named John Griffin, who was brought into the hospital on the 11th instant ; and both cases well illustrate the clinical history and the main points connected with the pathology of hooping-cough.

The great and all-absorbing feature of the disease is, that it is essentially "a cough;" it begins with a cough, and, until the malady is fairly worn out, this symptom continues to be present in a more or less severe form. The cough consists of a rapid succession of the most powerful expiratory efforts; so powerful, that some parts of the lungs must be as completely emptied of air by them as the mechanical arrangements of these organs will admit of. It appears probable that some lobules get quite emptied of air in this way, and thus is explained the condensation of portions of the lungs, occurring in this disease; but to this point I shall refer presently.

The violence of the cough, also, often excites vomiting, by means of which the stomach gets completely emptied of its contents, the paroxysm is stopped, and the patient experiences perfect relief for the time; if he be a child, he returns to his play, or, if seized while eating, he goes back to his food, (the appetite being generally very good, oftentimes voracious,) until, by and by, a fresh accumulation takes place in the stomach, the same train of expiratory efforts and vomiting is again gone through, and complete relief is again afforded.

There is one circumstance connected with this cough by which it differs from all other coughs, namely, that the series of expiratory efforts are so powerful, and expel the air so largely from the lungs, that there follows upon them a long protracted *inspiratory* act, which is accompanied by the well-known whooping sound, so highly characteristic of this disease. To explain the mode of formation of this peculiar and characteristic sound, let us briefly consider what the precise condition of the air passages is just at the moment of inspiration. In the forcible efforts to expel the air from the lungs by coughing, the larynx, trachea, and whole bronchial tree, are narrowed to their utmost extent, so that the inspiratory effort sometimes occupies a long time in the attempt to draw in the air, through the now constricted passages, while, above all, the glottis is closed, and a looker-on cannot but fear, that this spasmodic closure will produce the death of the patient; and sometimes, indeed, death does actually result from this cause, the closure of the glottis being quite as perfect as when an attempt is made to inhale carbonic acid. But at length the inspiratory effort succeeds, and, while it is taking place, the inward rush of the air through the narrowed and constricted fissure of the glottis produces the whooping sound which is truly pathognomonic of this disease.

Thus the whoop, which alarms the ignorant, is, to those who understand its mode of production, the signal of the child's safety, as the sound of thunder announces that the danger from the lightning-flash has passed.

There is no other symptom, besides this peculiar sound accompanying inspiration, which may be taken as certainly diagnostic of hooping-cough; and, when you are called to see a patient, supposed to be laboring under this complaint, it would not be safe to pronounce, that this is the malady, unless you have distinct evidence of this characteristic whoop. This peculiar sound, it is true, is sometimes imitated by other coughs, but it is never anything like so well defined, so far as my experience goes, in any other affection, as it is in this.

To be enabled to understand the pathology of hooping-cough, it is necessary to have some insight into the most important points in its clinical history.

Hooping-cough affects children mainly; indeed, it may be regarded as essentially a disease of childhood. There are some affections of the laryngeal organs, chiefly spasmodic affections of the muscles of the glottis, which appear to be in a great measure peculiar to this early period of life. To these affections, among which spasmodic croup, or laryngismus stridulus, occupies a prominent place, hooping-cough bears some analogy. It generally occurs at the early periods of childhood, but it is by no means limited to these ages; indeed, of the cases now in the hospital, the one patient is ten, the other fifteen years of age. It is rarely met with in adults, and it still more rarely attacks persons in the advanced periods of life; but that it sometimes does so, may be known from the fact, that, among the most recent cases of hooping-cough which I have witnessed, it occurred in the persons of an old married couple, aged eighty and seventy-two years respectively, and they got safely through this trying malady.

Another feature in the clinical history of hooping-cough is, that it is a contagious disease. This is exemplified by the cases now in the hospital, one of which I will briefly read to you: "Elizabeth Grffin, aged 15, admitted Dec. 21, 1852. She has always had very good health, and was never laid up until her present illness, which commenced six weeks previous to her admission, when her brother caught the hooping-cough, and has it severely." Now, this is the general history which we continually hear in cases of this complaint. One member of a family gets the disease, and it then spreads through the whole household, just like other contagious diseases, as measles, scarlatina, etc.

Hooping-cough has certain stages, which it is necessary we should describe more at length.

In the first stage, the symptoms are febrile and catarrhal, and the disease often passes for an ordinary cold. This state continues for ten or twelve days, and is then succeeded by the cough, the peculiar feature of which is, that it occurs in paroxysms, lasting some time, and following each other at variable intervals, and constituting the second stage of the disease; but, during the intervals between the paroxysms, the patient feels quite well, and one would hardly imagine there was anything the matter with him, except in the advanced periods of the complaint. The complete remission, which takes place, in many cases, between the paroxysms, is not the least curious feature in the disease. A minute or two after a violent fit of coughing, which you would imagine seriously jeopardised his life, you will see the child resume his play as merrily as if nothing had happened, and so he will go on.

The third stage varies considerably in different cases; the patient may either gradually recover, the symptoms becoming less and less marked, or certain changes may take place in the lungs and circulating system, which may lead to a fatal termination.

Let me direct your attention to these secondary changes, which occur in the lungs and vascular system, after the disease has lasted for some time. At first, the lungs are not at all affected; so that hooping-cough can no more be considered a disease of these organs, than can an aneurismal or other tumor pressing upon the vagus nerve, and, in this manner, exciting cough, be so regarded. After the cough has continued a long while, however, changes take place, as I just now stated, affecting the lungs and the general appearance of the patient. The countenance becomes full and bloated, and the capillaries distended, especially those of the conjunctivæ, which look watery and swollen; and some of these minute vessels often burst, giving rise to some chemosis. From this state of countenance, a practical eye can generally at once recognise the nature of the malady, under which the patient labors.

All these changes result from the circulation in the capillaries being retarded, in consequence of the violence of the cough. At the same time, and for a like reason, the pulmonary circulation becomes similarly affected; the secretion of the bronchial tubes becomes altered; these tubes pour fourth more freely than natural a watery mucus; the lungs become congested and œdematous; more or less crepitation is heard in different parts of these organs, according to the amount of fluid in the tubes or œdema present; and this crepitation is usually most audible towards the lower part, being sometimes more distinct in one lung than in the other. Both the patients in the hospital exhibit these changes to a considerable extent. The sound on percussion over the base of the lungs is duller than natural; and this arises mainly from the œdematous state of these organs, but in part, also, from the quantity of mucus present in the bronchial tubes, and from the expiratory efforts having emptied some lobules of air more completely than others, (some lobules being, perhaps, perfectly emptied in this manner, and consequently quite collapsed); and, lastly, from the altered bronchial secretion plugging up the entrance to one or more lobules, and in this way preventing the free ingress of air. This condition of lung, in which the ingress of air to certain portions is prevented, and of which certain other portions have also been completely emptied of the contained air, has been long known to pathologists under the name of "carnification." A carnified lung has a fleshy look, does not crepitate under pressure, and sinks in water; and this condition may be induced by anything which causes the complete expulsion of the air out of the lung, or which entirely prevents the ingress of air into a lung previously devoid of air, as one which has never respired; and it is best seen in the lung of a fœtus which has never breathed.—The most common cause of it, and that which, perhaps, developes it most completely, is the accumulation of fluid in the pleural cavity, by the pressure which it exerts on the adjacent lung. Carnification of the lung is carefully to be distinguished from hepatization. The former has nothing to do with inflammation, but merely consists in a condensation of the original pulmonary structure; the latter results from the effusion or exudation of an albumino-fibrinous material into the air-cells and finest bronchial tubes, by which the organ is rendered specially heavier.

It was formerly supposed, that lobular pneumonia took place in hooping-cough. That pneumonia, just as bronchitis, may occur in the course of hooping-cough is certain; but the signs which used to be considered as produced by lobular iuflammation, are, in reality,due to the simple exclusion of air from one or more lobules.

In the third stage of hooping-cough, if the case goes on favorably, there is a gradual abatement of the cough; the paroxysms become fewer and less severe, and the patient gradually returns to his normal state of health. But if, on the other hand, the progress is unfavorable, the symptoms become aggravated, the paroxysms more frequent, the bronchial tubes enlarged, the secretion of their relaxed mucous membrane increased, and, at length, the patient dies completely worn out and exhausted. If tubercles previously existed in the lungs in a quiescent condition, they are thrown into a state of activity, and symptoms of phthisis manifest themselves. Convulsions and coma, also, frequently accompany hooping-cough, especially in ill-nourished, badly-fed children, as the disease approaches its fatal termination. In these subjects, too, hooping-cough sometimes becomes complicated with an effusion of fluid into the lateral ventricles of the brain, and the phenomena of hydrocephalus are developed.

*Causes.*—In entering into the consideration of the causes of hooping-cough, one of the first questions that suggests itself is, whether the malady has its seat in the lungs. That hooping-cough is clearly no disease of the lungs, is shown both by its clinical history, and by the fact, that auscultation can detect nothing abnormal in the vioce or breath-sounds of a patient in the earlier stages of the complaint.

Its cause does not depend upon any peculiar state of the larynx or trachea; for there is no permanent affection of the voice, nor of the laryngeal muscles, nor of the glottis; nor are the symptoms such as diseases of the larynx or trachea usually give rise to.

Does the disease depend upon any morbid condition of the bronchial glands? The bronchial glands are often considerably enlarged without such a cough as the peculiar, paroxysmal one of this disease. Nor would the patient enjoy the complete freedom from distress which so often exists between the paroxysms of cough.

Having, then, set aside all these so-called causes of hooping-cough, the only supposition now left us as to the true cause of the disease is, that it depends upon some peculiar irritation of the vagus itself. In fact, hooping-cough is a special disease of this nerve, the irritation being quite as complete as when the exposed nerve is mechanically stimulated. But the cough differs from that which is produced by mechanical irritation of the nerve, in its coming on in paroxysms at longer or shorter intervals from each othe., the patient's health during the intervals being very good. This paroxysmal character of the disease, with the complete state of health in the intervals, except when the constitution or the lungs have bebecome damaged by the effects of the cough, associates hooping-cough with other diseases, the peculiar phenomena of which depend upon some poison in the blood, manifesting its presence by the specific action, which it exercises upon some particular tissue, and by the interference, which it seems to offer to the due performance of healthy function. For certain poisons undoubtedly appear to have a peculiar affinity for certain tissues; thus the poison of measles appears to have a special affinity for the mucous membrane of the bronchial tubes and bowels, that of scarlatina for the throat, and so on of the other acute specific diseases. In like manner the poison, which gives rise to the phenomena of hooping-cough, seems to have a peculiar affinity for the vagus nerve; but whether throughout the whole course of that nerve, at its centre or its periphery, it is impossible, in the present state of our knowledge, to affirm with any degree of accuracy. It is no valid objection to this view of the nature of the disease, to say, that after death, no structural alteration of the vagus can be distinguished, although Autenreith and others state, that in cases of hooping-cough examined after death, they have found the vagus in a congested condition. But congestion is much more frequently the effect, than the cause of a disease; and it may be especially so in this case. In many nervous affections, as for instance in those distressing cases of neuralgia, in which the most intense pain has existed during life, no appreciable morbid condition of the nerves supposed to be the seat of the pain can be detected on the most careful examination after death. The poison in hooping-cough, whatever it be, produces no structural lesion in the nerves, and leaves nothing behind it, of which our senses can take cognizance.

Hooping-cough, then, as far as present knowledge enables us to speak, is a disease which runs a certain course, can be communicated from one person to another, and is probably due to the influence of a poison which gets into the system, and produces its local manifestations on the vagus nerve. It is not an inflammatory affection of any part, being simply dependent on a morbid state of the blood, caused by the introduction into it of some poison from without; and whatever inflammations may occur in the course of it must be regarded in the light of complications of the disease.

Having advanced this view of the nature of the disease, let me make a few observations upon its rational treatment, as founded upon these opinions.

Assuming hooping-cough to be a disease, depending on the presence of a morbid poison in the blood, (which is the most reasonable view of its pathology,) to cure the affection perfectly, we ought to find an antidote for the poison which produces it. If we could find some material which, when introduced into the system after it had received the poison, would neutralise that poison, then we should have the same power over the malady, as we now possess over intermittent fever, which, as you know, is also a paroxysmal disease, depending on the presence of some morbid poison in the system, and for which an antidote has been found in bark. But since, unfortunately, no antidote for hooping-cough has as yet been discovered, it should not be our practice to look on in silence, and let the patient cough it out; but our aim should be to find the means of guarding him against the bad consequences of the cough, and to protect him from all those complications, to which I referred at the commencement of the lecture.

As the disease does not consist in an inflammatory condition of any part, we may at once dismiss all so-called antiphlogistic plans of treatment. That plan indeed, has had a fair trial; and if it had any real power over the disease, we should have, long ere this, accumulated abundant evidence to prove its superiority. The tendency of all the usual antiphlogistic measures is to weaken the nutrition of the lungs and the nervous system, and to impoverish the blood; to reduce the quantity of its coloring-matter, to favor the accession of convulsions, and by the watery parts of the blood filtering through the walls of the blood vessels, to promote the tendency to hydrocephalus.

The first point in the treatment is, carefully to guard the patient against the occurrence of bronchitis and pneumonia, as complications of the disease. Now, there is nothing which is so fertile a cause of bronchitis, as the admission of cold air to the bronchial mucous membrane. Consequently, the patient should be kept in a well regulated temperature; if his illness occur in the winter, he should stay in-doors, in a roomy, well ventilated apartment, which is not too warm but of a uniform heat. He should be kept in this apartment, and not allowed to run about the house into rooms, or upon lobbies or staircases, which must present great variety of temperature. Early and close attention to this maintenance of a uniform temperature of the atmosphere in which the child resides, may save much subsequent mischief.

The second point is to uphold the general nutrition—to keep the patient well nourished. I do not mean, that the patient should be crammed or over-fed, but that his diet should be well regulated, and sufficient food of all kinds supplied, not only to satisfy the appetite, but also—and what is far more important—the real wants of the system. On this account, I object to keep children in this disease without animal food, as some so much insist on, though why they do so I cannot tell; for meat in regulated quantities, and properly masticated, is more easily digested than almost anything else; and it differs from other alimentary substances, in the fact that its digestion consists in a simple process of solution in the stomach.

Another practice which exercises a most favorable influence on the nervous system, (and it is this that we must look to after all,) is sponging the chest with *cold water* once or twice a day. The parents of weakly, delicate children often object to this plan of treatment; but by ordering a little spirit to be mixed with the water, you not only may overcome their scruples, but in giving a stimulating quality to the application increase its efficacy. This sponging of the back and front of the chest, night and morning, exercises a bracing and tonic influence on the nerves, and in this way acts very beneficially in this disease. Spirituous embrocations often do good in a similar manner.

In a large number of cases, one can get on very well without having recourse to drugs. Those which you will find most useful, and which I would recommend to your notice, are sedative and anti-spasmodic remedies, in virtue of the power which they possess in allaying irritability of the nervous system generally, such as the various preparations of opium, henbane, conium, belladona, and hydrocyanic acid. The non-nauseating expectorants,, such as chloric ether, ammonia, and perhaps senega, may be also used; and astringents, to check excessive bronchial secretion, such as alum, sulphate of zinc, tannic and gallic acids, are sometimes necessary.—But you must bear in mind, that such remedies should be used with caution, especially opiates, which in infancy and childhood are at all times to be given with great care, and more particularly if the lungs have become congested. The drugs which I would recommend you to avoid, are those which have a depressing and lowering tendency, such as tartar emetic and ipecacuanha. Many children, I am quite satisfied, while suffering from hooping-cough, have died from the too free and slovenly exhibition of these emetics.

If I had an opportunity of treating hooping-cough on the large scale, I would, in cases in which the paroxysms are very frequent and very severe, and when, as yet, the lungs are free from congestion, but not otherwise, give a fair trial to the careful inhalation of chloroform, with the view of endeavoring to cut short the paroxysm. We know that we can arrest the paroxysm of asthma in this way; why, then, should we not be able to do the same with that of hooping-cough? I have also known laryngismus stridulus relieved by the use of chloroform; and it is now well proved that other convulsions of children may be checked by its means.

In the cases of delicate children, where there is great reason to fear that damage may be done to the lungs by the cough, this practice may prove very useful. But, with reference to the administration of chloroform, this fact should always be borne in mind, and it cannot be too frequently reiterated, that due provision should be made for the simultaneous free admission of air, along with the vapor of chloroform. There is no point upon which some men seem to be more foolhardy than on this one; and it is by the neglect of attending to this, that the reputation of one of the most valuable remedies that has ever been applied to the relief of human suffering may be seriously damaged. I do not advise you to give chloroform, so as to produce its full effect; it may be inhaled in small doses of ten or fifteen minims, which may be repeated at intervals, according to the severity of the paroxysms. When children are already in an exhausted and very depressed state, chloroform ought not to be administered by inhalation, or it should be given only in the smallest quantities.

Another remedy in the treatment of hooping-cough, to which I should very much like to give a fair trial, is the application of cold water, on the splashing plan, two or three times dialy, with or without the inhalation of chloroform. Such a practice must be pursued with proper precautions; first, to maintain a warm temperature of the room in which it is done; and, secondly, to have the water thrown over the child rapidly, and not so as to wet the head. To let the back and chest receive the brunt of the splash. These measures combined, would tend to diminish the severity of the paroxysms, ward off the occurrence of bronchitis and pneumonia, as complications of the disease, promote the general nutrition, stimulate the nervous system, and thus protect the patient from the damaging effects of the cough.

